# Supplementary material for: Auditor models to suppress poor artificial intelligence predictions can improve human-artificial intelligence collaborative performance
Source: J Am Med Inform Assoc. 2026 Jan 13;33(3):621–31. doi: 10.1093/jamia/ocaf235 (PMC12981670; doi:10.1093/jamia/ocaf235)

Supplementary Materials

**Title:** Auditor Models to Suppress Poor AI Predictions Can Improve Human-AI Collaborative Performance

**Authors:**

Katherine E. Brown, PhD^1^

ORCID: <https://orcid.org/0000-0003-4443-8541>

Jesse O. Wrenn, MD, PhD^1,2^

ORCID: <https://orcid.org/0000-0002-9869-9328>

Nicholas J. Jackson, BS^1^

ORCID: <https://orcid.org/0000-0001-6763-599X>

Michael R. Cauley, PhD^1^

ORCID: <https://orcid.org/0000-0003-3835-2725>

Benjamin Collins, MD, MS^1^

ORCID: <https://orcid.org/0000-0002-6884-3819>

Laurie Lovett Novak, PhD, MHSA^1^

ORCID: <https://orcid.org/0000-0002-0415-4301>

Bradley A. Malin, PhD^1,3,4^

ORCID: <https://orcid.org/0000-0003-3040-5175>

Jessica S. Ancker, MPH, PhD^1^

ORCID: <https://orcid.org/0000-0002-3859-9130>

**Affiliations:**

^1^Department of Biomedical Informatics, Vanderbilt University Medical Center, Nashville, Tennessee

^2^Department of Emergency Medicine, Vanderbilt University Medical Center, Nashville, Tennessee

^3^Department of Biostatistics, Vanderbilt University Medical Center, Nashville, Tennessee

^4^Department of Computer Science, Vanderbilt University, Nashville, Tennessee

Corresponding Author: Katherine E. Brown, PhD, Department of Biomedical Informatics, Vanderbilt University Medical Center, Suite 1475, 2525 West End Avenue, Nashville, TN 37203, USA; [katherine.brown@vumc.org](mailto:katherine.brown@vumc.org)

**S.1 Additional Dataset and Model Information**

Table S1. Enumeration of features in the MIMIC-IV and MIMIC-IV ED datasets originating from Beth Israel Deaconess Medical Center. For values in between angled brackets (<…>), there is a single feature per value. Feature name, type, description, and usage per task is included.

| Feature Name | Type | Description | Clinical Task Used |
| --- | --- | --- | --- |
| No. Days in the ED in the past <1/3/730/90/365> days | Integer | Number of days patient has visited the ED in the last <1/3/7/30/90/365> | ED Triage  ED Discharge |
| No. Days as an Inpatient in the Hospital in the past <1/3/730/90/365> days | Integer | Number of days patient has been admitted to the hospital in the last <1/3/7/30/90/365> | ED Triage  ED Discharge |
| No. Days in the ICU in the past <1/3/730/90/365> days | Integer | Number of days patient has been admitted to the ICU in the last <1/3/7/30/90/365> | ED Triage  ED Discharge |
| Age | Float | Age of patient | ED Triage  ED Discharge |
| Gender | Categorical | Male/Female | ED Triage  ED Discharge |
| Race | Categorical | Race of patient | ED Triage  ED Discharge |
| Arrival Transport | Categorical | Means of arrival for patient | ED Triage  ED Discharge |
| Triage <Temperature/Heart Rate/Respiratory Rate/O2 Saturation/Systolic BP/Diastolic BP/Pain Level> | Continuous | Specified vital sign at triage | ED Triage  ED Discharge |
| Average <Temperature/Heart Rate/Respiratory Rate/O2 Saturation/Systolic BP/Diastolic BP/Pain Level> | Continuous | Average of specified vital sign during ED visit | ED Discharge |
| Last <Temperature/Heart Rate/Respiratory Rate/O2 Saturation/Systolic BP/Diastolic BP/Pain Level> | Continuous | Last measurement of specified vital sign during ED visit | ED Discharge |
| Emergency Severity Index | Categorical | 1 (most severe), 2, 3, 4, 5 (least severe) | ED Triage  ED Discharge |
| Chief Complaint Present | Binary | Is the specified chief complaint present in this patient? (0: No/1: Yes) | ED Triage  ED Discharge |
| Charleston Comorbidity Index | Binary | Is the specified comorbidity present? (0: No/1: Yes) | ED Triage  ED Discharge |
| Elixhauser Comorbidity Index | Binary | Is the specified comorbidity present? (0: No/1: Yes) | ED Triage  ED Discharge |

Table S2. Enumeration of features in the datasets derived from Vanderbilt University Medical Center’s Clarity database. For values in between angled brackets (<…>), there is a single feature per value. Feature name, type, description, and usage per task is included.

| Feature Name | Type | Description | Clinical Task Used |
| --- | --- | --- | --- |
| No. Days in the ED in the past <1/3/7/30/90/365> days | Integer | Number of days patient has visited the ED in the last <1/3/7/30/90/365> | ED Triage  ED Discharge |
| No. Days as an Inpatient in the Hospital in the past <1/3/7/30/90/365> days | Integer | Number of days patient has been admitted to the hospital in the last <1/3/7/30/90/365> | ED Triage  ED Discharge |
| No. Days in the ICU in the past <1/3/7/30/90/365> days | Integer | Number of days patient has been admitted to the ICU in the last <1/3/7/30/90/365> | ED Triage  ED Discharge |
| Age | Continuous | Age of patient | ED Triage  ED Discharge |
| Gender | Categorical | Male/Female | ED Triage  ED Discharge |
| Race | Categorical | Race of patient | ED Triage  ED Discharge |
| Arrival Transport | Categorical | Means of arrival for patient | ED Triage  ED Discharge |
| Triage <Temperature/Heart Rate/Respiratory Rate/O2 Saturation/Systolic BP/Diastolic BP/Pain Level> | Continuous | Specified vital sign at triage | ED Triage  ED Discharge |
| Average <Temperature/Heart Rate/Respiratory Rate/O2 Saturation/Systolic BP/Diastolic BP/Pain Level> | Continuous | Average of specified vital sign during ED visit | ED Discharge |
| Last <Temperature/Heart Rate/Respiratory Rate/O2 Saturation/Systolic BP/Diastolic BP/Pain Level> | Continuous | Last measurement of specified vital sign during ED visit | ED Discharge |
| Emergency Severity Index | Continuous | 1 (most severe), 2, 3, 4, 5 (least severe) | ED Triage  ED Discharge |
| Chief Complaint Present |  | Is the specified chief complaint present in this patient? (0: No/1: Yes) | ED Triage  ED Discharge |
| Elixhauser Comorbidity Index | Binary | Is the specified comorbidity present? (0: No/1: Yes) | ED Triage  ED Discharge |

Table S3. Distribution of outcomes and majority and minority classes of demographics in the MIMIC-IV data across both tasks.

|  |  | ED Triage | | ED Discharge | |
| --- | --- | --- | --- | --- | --- |
|  | Ground Truth | No Negative Outcome | Negative Outcome | Not Readmitted within 30 days | Readmitted within 30 days |
| Age | 18-49 | 163,574 | 4,559 | 104,275 | 51,203 |
|  | >= 50 | 204,569 | 20,874 | 73,862 | 133,805 |
| Race | White | 197,421 | 15,635 | 85,619 | 110,709 |
|  | Not White | 170,722 | 9,798 | 92,518 | 74,299 |
| Gender | Male | 165,434 | 13,652 | 74,251 | 89,716 |
|  | Not Male | 202,709 | 11,781 | 103,886 | 95,292 |
|  | Total | 368,143 | 25,433 | 178,137 | 185,008 |

Table S4. Distribution of outcomes and majority and minority classes of demographics in the VUMC data across both tasks.

|  |  | ED Triage | | ED Discharge | |
| --- | --- | --- | --- | --- | --- |
|  | Ground Truth | No Negative Outcome | Negative Outcome | Not Readmitted within 30 days | Readmitted within 30 days |
| Age | 18-49 | 32,389 | 1,011 | 20,835 | 14,733 |
|  | >= 50 | 23,961 | 1,456 | 9,958 | 13,297 |
| Race | White | 35,745 | 1,785 | 18,564 | 18,968 |
|  | Not White | 20,605 | 682 | 12,229 | 9,066 |
| Gender | Male | 26,511 | 1,452 | 13,230 | 14,733 |
|  | Not Male | 29,839 | 1,015 | 17,563 | 13,301 |
|  | Total | 56,350 | 2,467 | 30,793 | 28,034 |

Table S5. Gradient-boosting tree (GBT) parameters for each dataset and task. Tree depth is the maximum number of nodes from the root to the leaf of a tree in the GBT ensemble. The L2 regularization coefficient indicates the amount of regularization placed on each tree of the ensemble.

| Institution | Task | Tree Depth | L2 Regularization Coefficient |
| --- | --- | --- | --- |
| MIMIC-IV | Triage | 6 | 9 |
| MIMIC-IV | Discharge | 10 | 5 |
| VUMC | Triage | 4 | 9 |
| VUMC | Discharge | 6 | 1 |

**S.2 Results of Sensitivity Analysis**

We performed a sensitivity analysis to understand the impact of varying the *in silico* simulation of how humans would likely respond to AI predictions. We varied the threshold at which AI predictions were randomly accepted by clinicians for patients who do not meet the following criteria: the patient was elderly, had polypharmacy (requires multiple medications), has five or more chronic conditions, or had a severe chief complaint. In our primary experiments, we assume a 20% acceptance rate for patients outside of these criteria. This sensitivity analysis considers varying this acceptance rate to 0%, 50% and 80%. Each experiment was run 15 times for both prediction tasks at VUMC and BIDMC. We present the fairness-utility tradeoff visualizations and heatmaps of Mann-Whittney U test for statistical significance.

Figure S1. Sensitivity analysis results for triage task at VUMC and BIDMC for an AI acceptance rate of 0.0


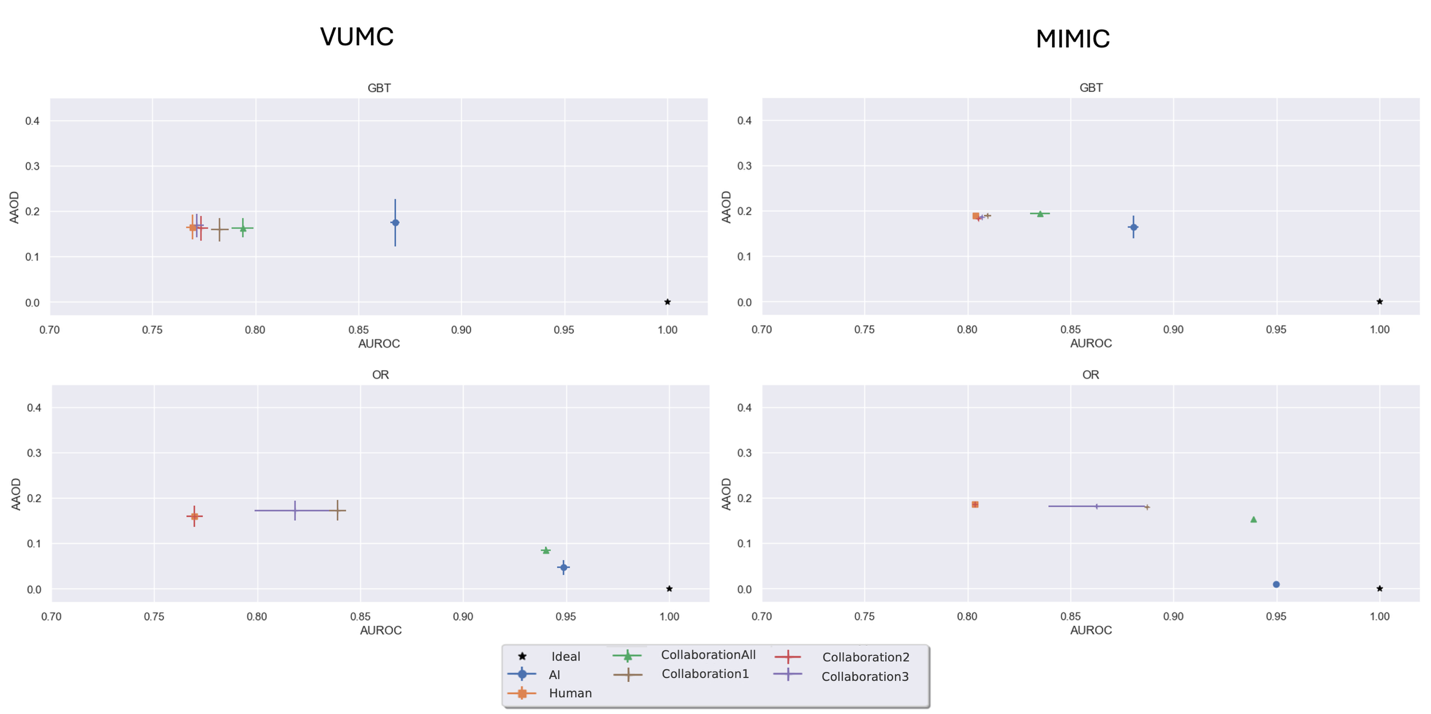

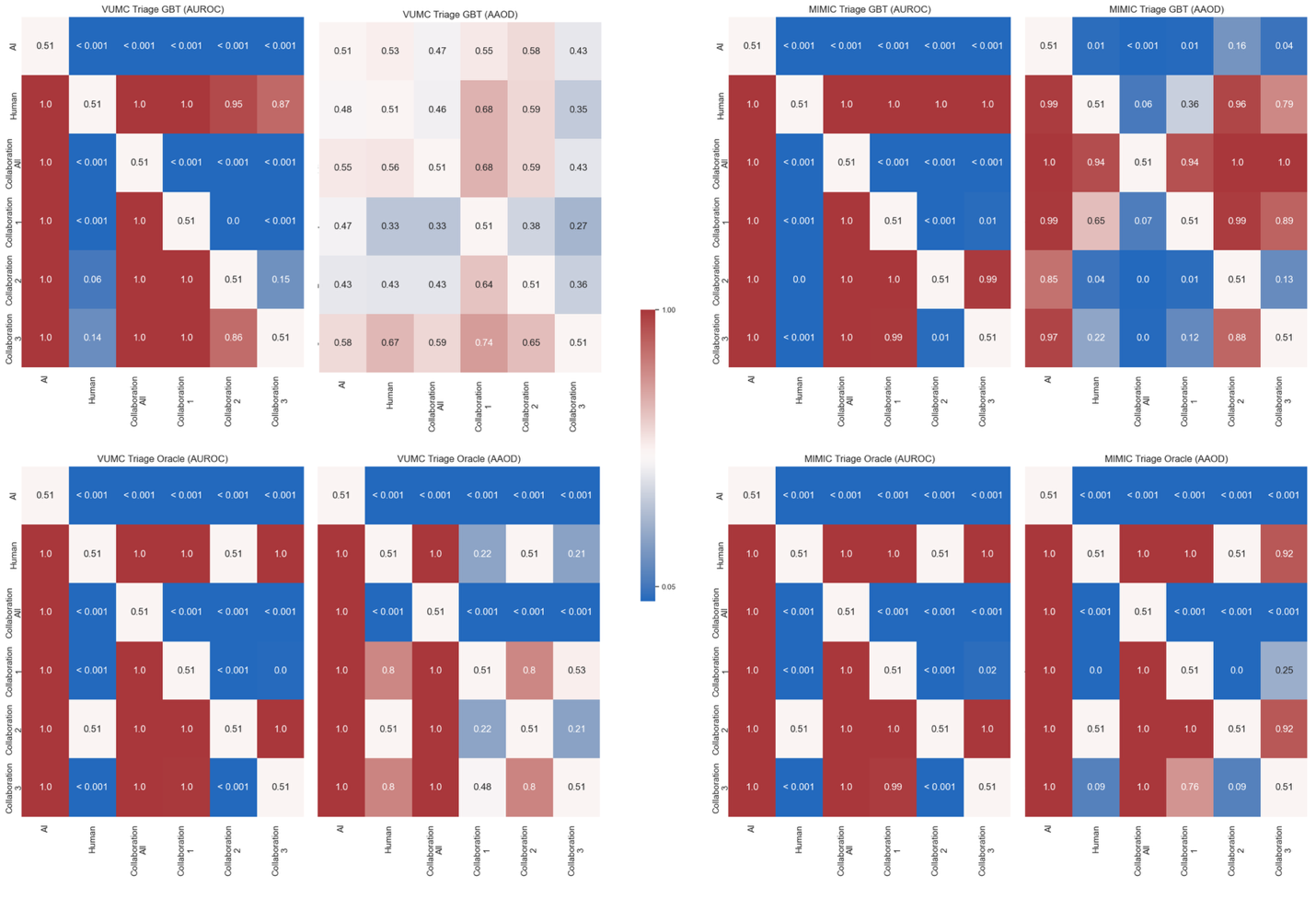


Figure S2. Sensitivity analysis results for triage task at VUMC and BIDMC for an AI acceptance rate of 0.5.


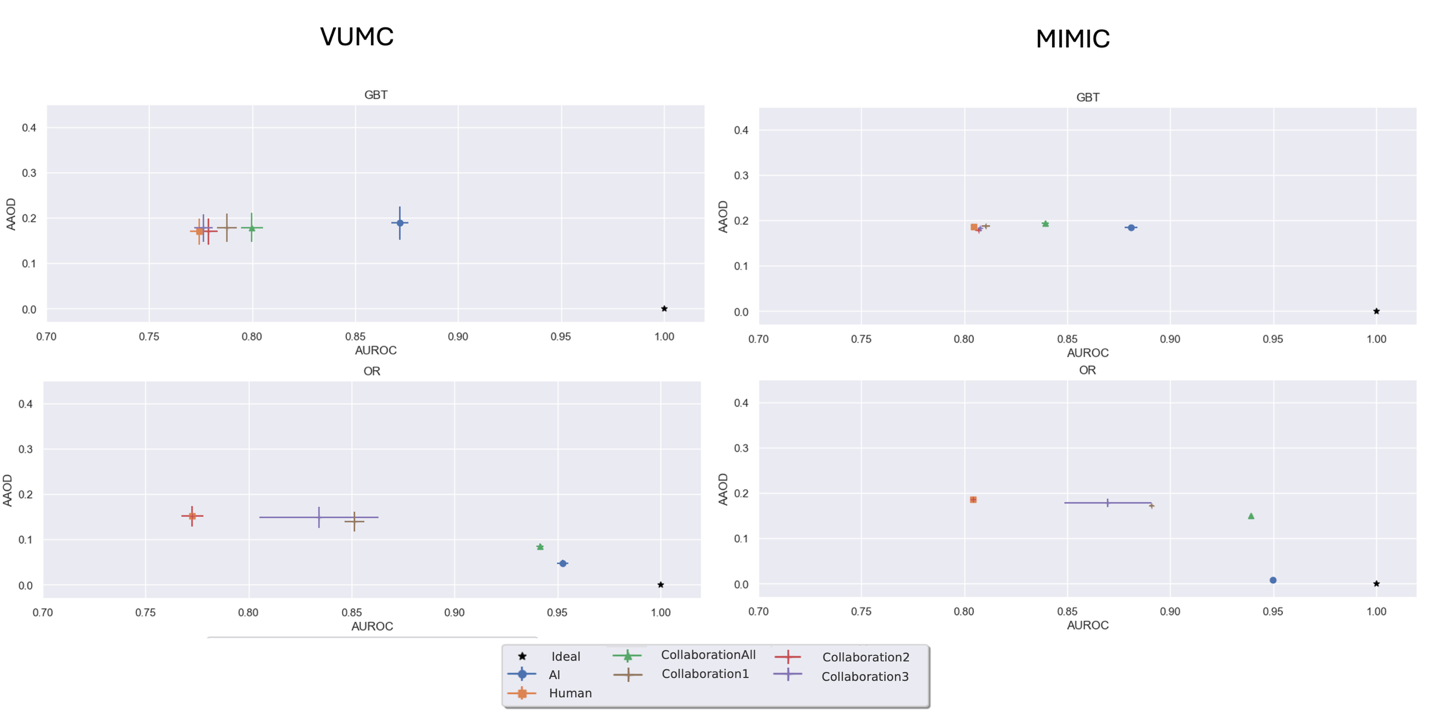

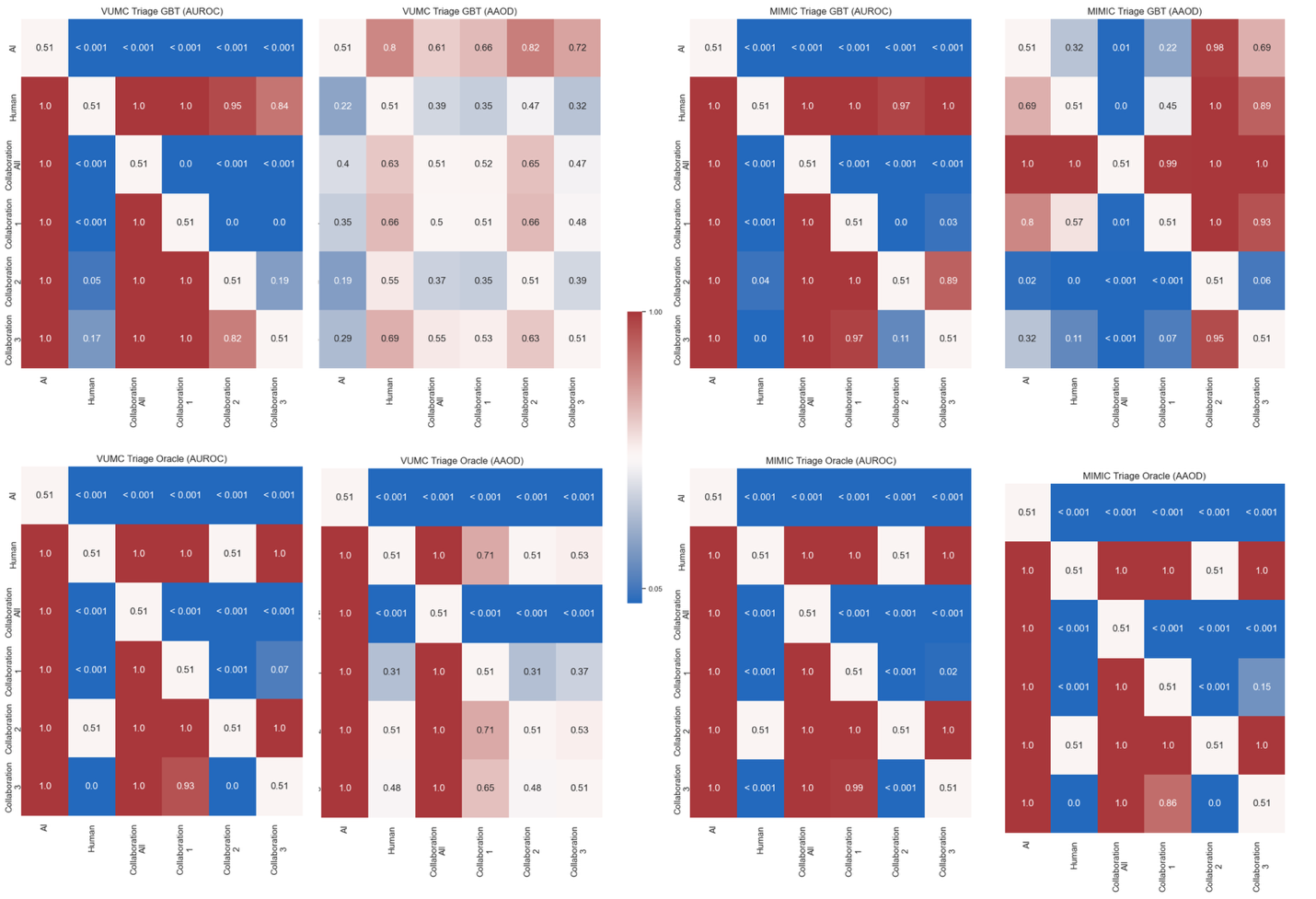


Figure S3. Sensitivity analysis results for triage task at VUMC and BIDMC for an AI acceptance rate of 0.8.


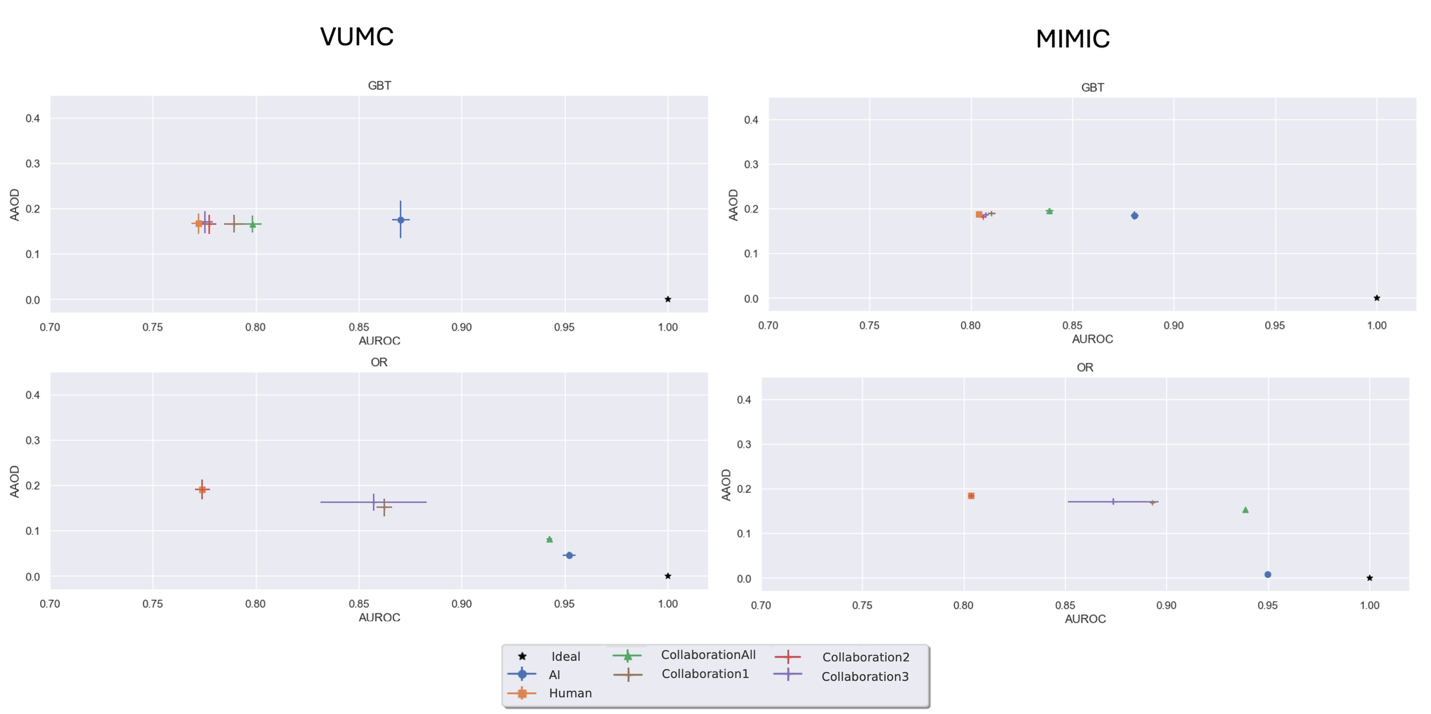

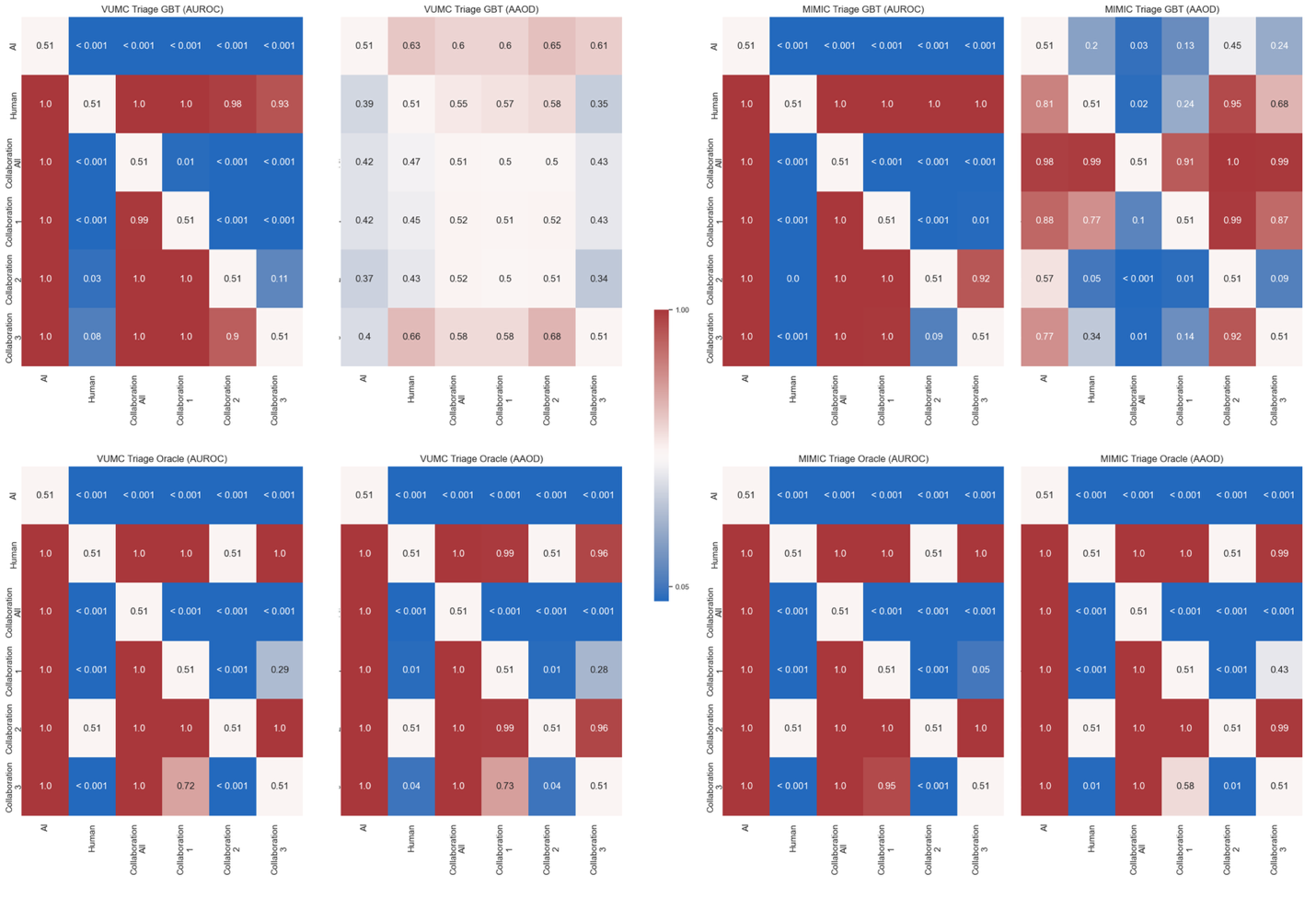


Figure S4. Sensitivity analysis results for readmission task at VUMC and BIDMC for an AI acceptance rate of 0.0.


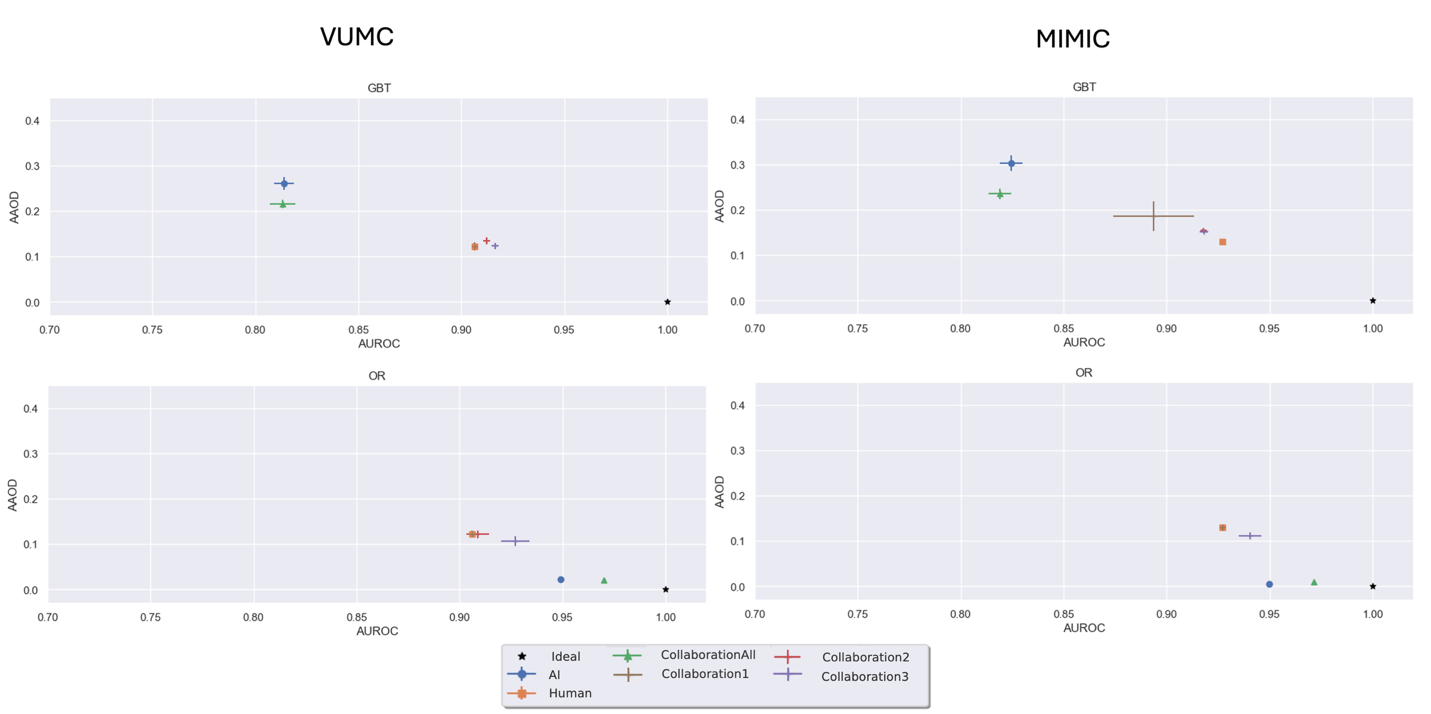

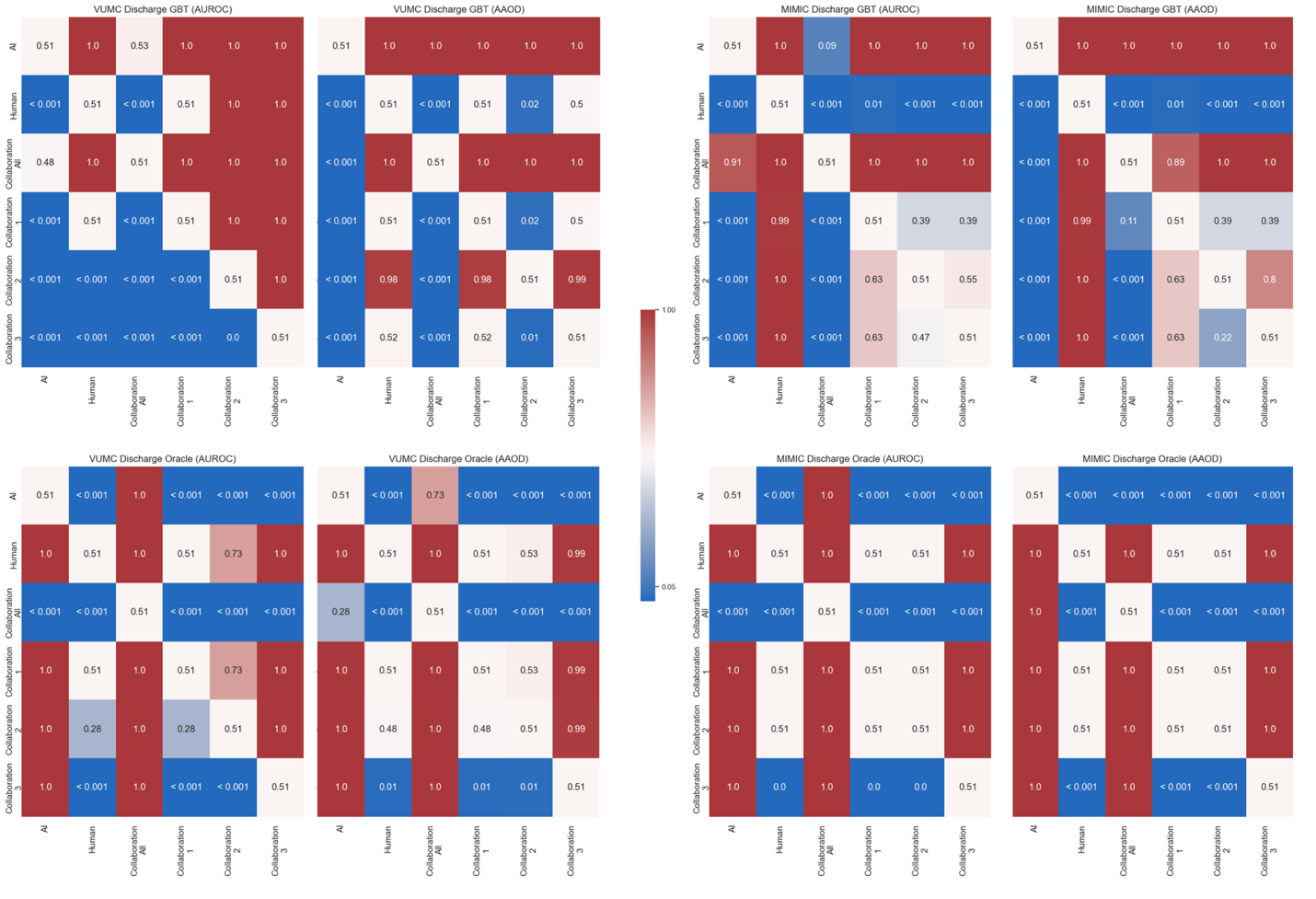


Figure S5. Sensitivity analysis results for readmission task at VUMC and BIDMC for an AI acceptance rate of 0.5.


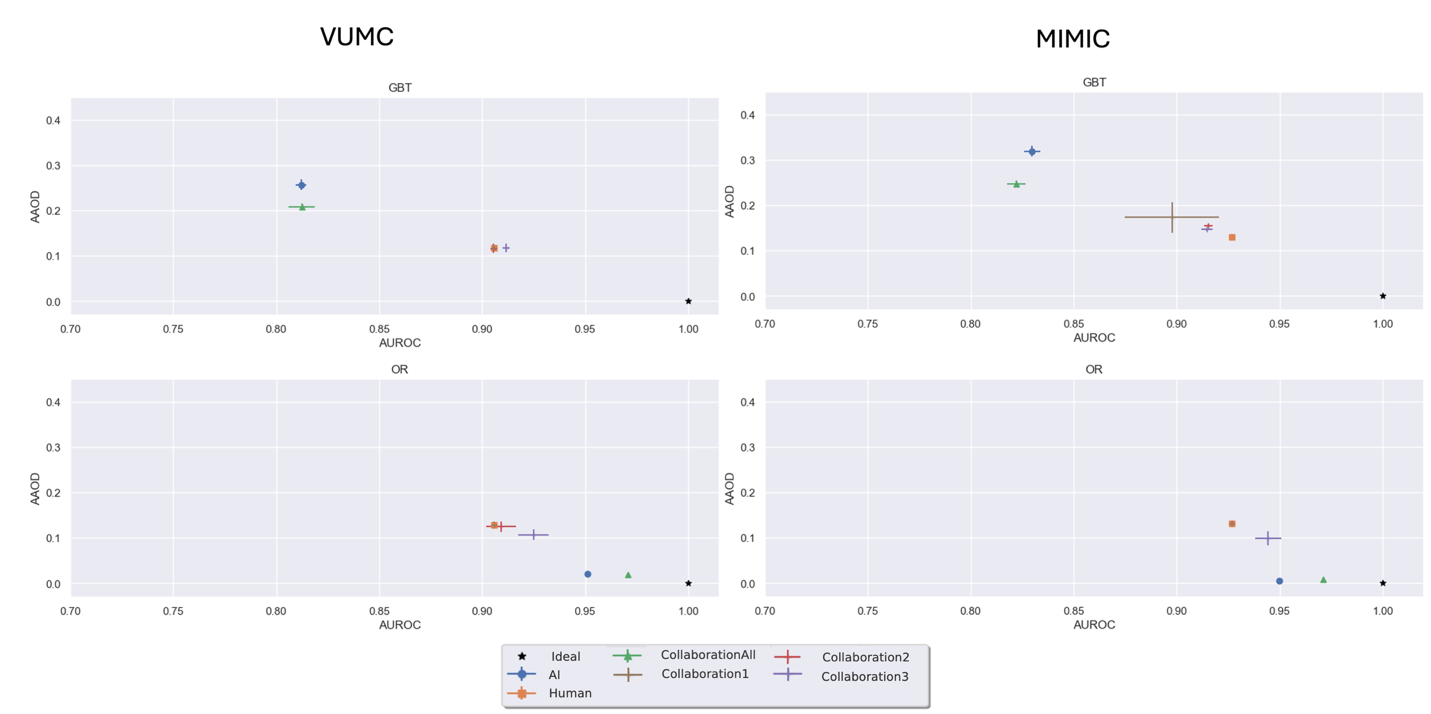

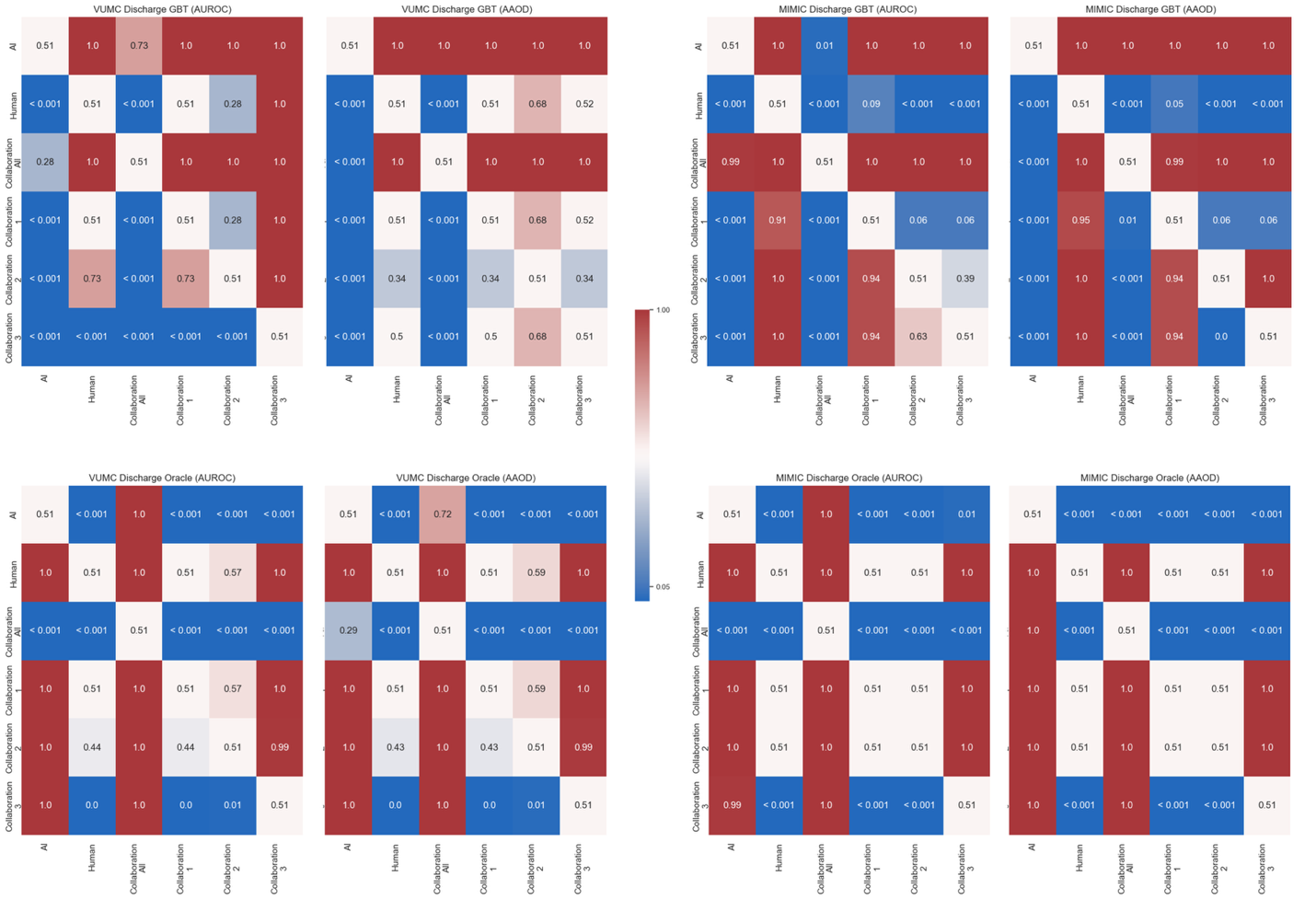


Figure S6. Sensitivity analysis results for readmission task at VUMC and BIDMC for an AI acceptance rate of 0.8.


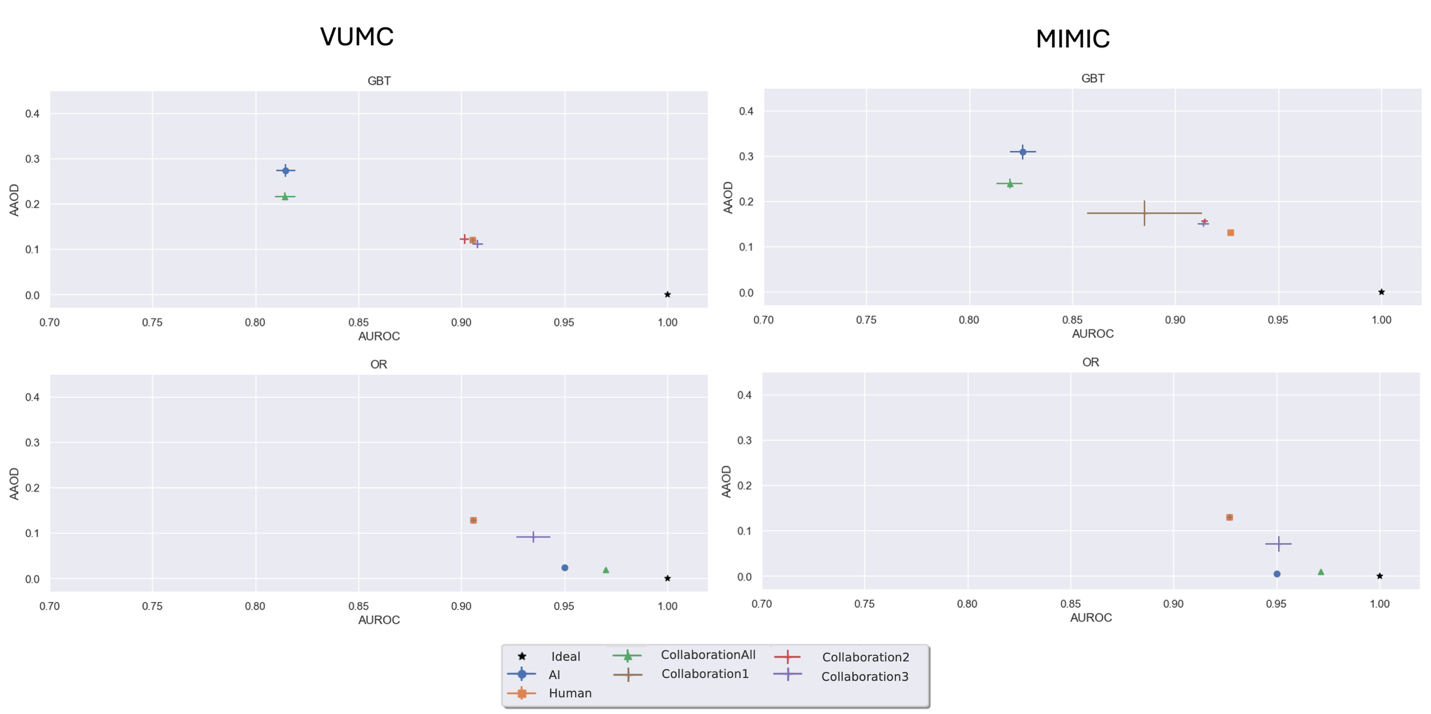

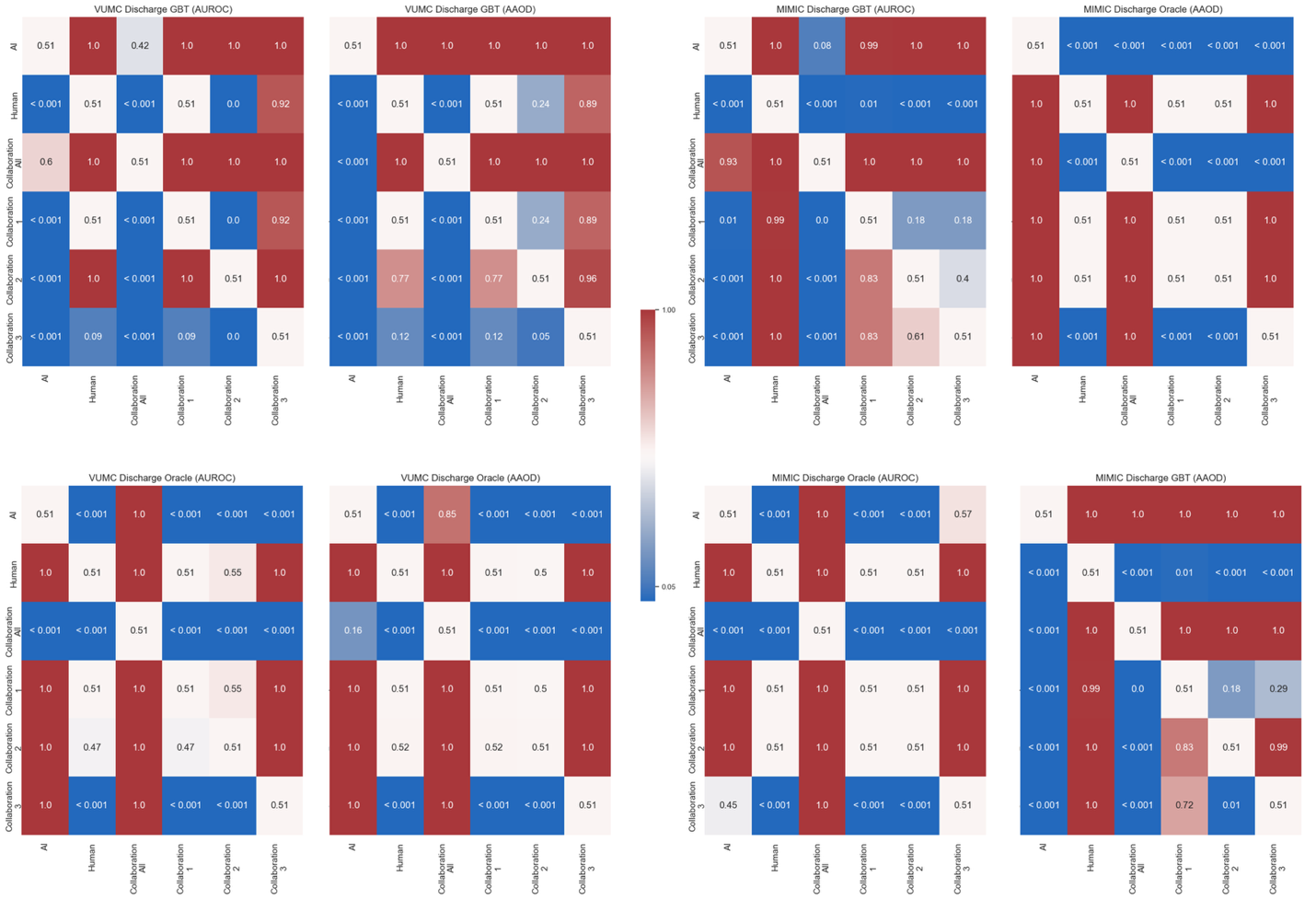

Supplement: ocaf235_Supplementary_Data [file ocaf235_supplementary_data.docx]
